# Substance of a Clinical Lecture on a Case of Hydrophobia, Delivered at the Charing Cross Hospital, Monday, November 24, 1834; to Which Are Appended the Particulars of Another Case Admitted into the Hospital, October 21, 1834

**Published:** 1835-04-01

**Authors:** 


*Substance of a Clinical Lecture on a Case of Hydrophobia, delivered at the Charing Cross Hospital, Monday, November* 24, 1834; *to which are appended the Particulars of another Case admitted into the Hospital, October* 21, 1834. By T. J. Pettigrew, F.R.S. &c., Surgeon to the Hospital.—London, 1834. 8vo. pp. 35.

The subject of this lecture was a shopman, æt. eighteen, who was bitten by a mad dog, between the 21st and 28th September, 1834; and the symptoms of hydrophobia began on the 18th of November. Mr. Pettigrew observes, that this is the period after the bite at which the disease most commonly commences.

"In the majority of cases, the disease manifests itself within two months. Dr. Hamilton constructed a table (which I am at present engaged in extending, and the result of which I shall lay before you at a future time,) from which it appears that the disease seldom appears before the nineteenth day or after the eighteenth month. In 131 cases, the number from the eighteenth to the thirtieth day are only 17; from the thirtieth to the fifty-ninth, 63; from two to three months, 23; from three to four months, 9; five months, 2; five months and eleven days, 1; six months, 1; seven months, 1; eight months, 2; between eight and nine months, 1; nine months, 2; eleven months, 1; fourteen months, 1; eighteen months, 2; nineteen months, 1; which perhaps is, of well authenticated cases, the longest period known. All stated to have occurred beyond this time are probably fabulous, or not depending upon the bite to which they have been referred. The ancients generally ascribed forty days as the time at which the disease would become apparent, and subsequent observation has confirmed the remark." (P. 22.)

The patient survived the attack three days, the last one of which was passed in the Charing Cross Hospital. The treatment consisted chiefly of tobacco enemata. The following were the post-mortem appearances:

"*Spinal Chord.* The theca was of the natural appearance; no fluid within it, and no particular vascularity upon the surface of the chord itself, the medulla of which presented its natural whiteness.

"*Head.* The dura mater adhered with great firmness to the skull. The longitudinal sinuses were empty: a small quantity of blood was seen in a fluid state in some of the large veins leading to the sinuses. The hemispheres of the brain had a milky white appearance, upon the removal of the dura mater, and this was observed to be greatest in the intergyral spaces between the convolutions: the general milkiness of the membranes disappeared in some degree upon exposure to the air. The membranes were much less injected with blood than could be expected. The substance of the brain was very firm, and less vascular than ordinary; there were fewer bloody points from divisions of vessels observed than usual. A quantity of fluid, amounting to between two and three drachms, was found in the lateral ventricles: it was not at all bloody. The plexus choroides was turgid with venous blood, and the vessels in the left ventricle were much fuller than those of the right. The pineal gland contained no sabulous matter, but was very tough in its substance, which did not break down under the pressure of the fingers, as usual. The greatest vascularity observed throughout the brain was of the pia mater, over the pons Varolii, and medulla oblongata. Here the vessels were highly injected with arterial blood, particularly on the right side, and they were very strongly adherent to the parts beneath. The membranes over the optic nerves and the anterior crura cerebri were also very vascular. The absence of vascularity in the brain generally was remarkable, and not a drop of fluid was found at the basis. The lateral sinuses, like the longitudinal, were empty.

*Neck.* The muscles were dark coloured, and fuller of blood than usual. The tongue had its papillæ very large, particularly at the root; there were no pustules about the frænum. The tonsils were much enlarged, but not vascular. The pharynx and œsophagus were throughout perfectly natural; there were not the slightest appearances of inflammation in any part. The larynx and trachea were also free of any marks of vascularity, excepting at the bifurcation of the latter at its entrance into the lungs, where it was slightly reddened. The inner surface of the larynx and trachea were smeared with a dark-coloured fluid, which appeared to be a portion of a dark bilious fluid, a small quantity of which was found in the stomach, and of which a considerable quantity had been vomited up prior to death.

" *Thorax.* Not a drop of effusion was contained in either cavity of the chest. All the viscera had their natural appearance; the lungs contained air, and were rather remarkable for the very small quantity of blood in them. There were some adhesions between the pleuræ on the left side, but they were not recent. The phrenic nerves and the diaphragm presented their usual natural appearance; the heart was rather more fatty than is usually found in persons of so early an age. The pericardium contained about half an ounce of light straw-coloured fluid. The left ventricle was empty, firm, and thick, and its substance of a dark colour; the right ventricle had some small portions of coagula. The large vessels presented no marks of increased action; their inner surfaces were quite white. The great sympathetic nerve was perfectly healthy.

*Abdomen.* The liver was natural, but the gall-bladder was distended with a bile, perfectly black: it had communicated no tinge to the surrounding parts. The stomach was very much contracted; and, upon opening it, it was found to contain about four ounces of a greenish-coloured fluid. The rugæ were very strongly marked, and the glands about the cardia and pylorus were more conspicuous than usual, and contained a whitish coloured deposit, giving a strumous appearance to them. This conjecture is supported by the enlarged state of the tonsils, a much enlarged condition of the mesenteric glands, and also an increase of firmness in the pancreas. No abrasions or extravasations of any kind were observable in the stomach; neither was there any appearance of vascularity to be seen, except towards the pylorus, where a slight redness was discernible. The intestines were distended with air, and presented a very dry appearance: they contained no feculent matter. The small intestines presented no particular vascularity, and there were no spots of discoloration. The whole of the descending colon and the rectum were powerfully contracted, but not diseased in their structure; there was also a contraction in the centre of the transverse arch of the colon.

"The kidneys were healthy, but the urinary bladder was very firmly contracted, and as hard to the feel as a dense fleshy mass; the muscular fibres were observable through the peritoneal covering firmly contracted. The penis was observed to be in a state of tension, that may be considered as semi-priapism.

"*Hand.* The punctures on the palm of the right hand were examined, and the nerves of those parts traced, but no appearance of inflammation or disorder of any kind was apparent." (P. 19.)

In the other case, the patient was a man, æt. forty-seven, who had been bitten by a cat, five weeks before the appearance of the symptoms. He was admitted into the Charing-cross Hospital, on the 21st of last October, and died on the following afternoon. Tobacco enemata and strychnia pills were the chief remedies employed. The spinal chord was not diseased, and the post-mortem examination presented but little worthy of note. The following is a part of it.

"*Abdomen.* The stomach contained a little mucus: it was considerably inflamed near its cardiac extremity, and at the lower part of its smaller end the surface was abraded. This did not appear to have arisen from the action of the gastric juice, for the vessels were distinctly visible, ramifying minutely at this spot. The duodenum was strongly tinged with bile, and slightly inflamed. The jejunum was inflamed, but the remainder of the small intestines, and the larger ones, were quite healthy. The liver was indurated, granular in its appearance, and had adhesions to the diaphragm: none of these appearances could be regarded as recent. The gall-bladder was full, and its ducts pervious. The spleen was enlarged, and gorged with blood. The kidneys were more injected with blood than usual, and vessels were observable ramifying on the pelvis of them. The bladder was found strongly contracted.

"*Neck and Thorax.* The trachea was inflamed, particularly between the rings: a similar condition was found to exist in the bronchiæ and their termination. The pleura was healthy, but contained a pint of fluid. The lungs were gorged with serum, and blood and sputa. In the bronchial plexus of nerves there appeared no change. The substance of the heart was softer than natural, and the right side contained several coagula. The left auricle was of a deeper colour than the left [sic]. The aorta appeared of an uniformly high red colour, which increased in depth as it approached nearer the heart." (P. 34.)

Our author still thinks that tobacco clysters deserve further trial, for he does not allow that they have yet had a fair one. In the case of Grindley, the shopman, the malady was too far advanced; and the older patient, in addition to this disadvantage, had a frame broken down by organic disease. "Twenty-six years since my excellent friend Dr. Clutterbuck had a case of hydrophobia, in a delicate child, to whom, in an advanced state of the disease, an injection of tobacco was administered, with the view of allaying the violent spasms operating upon the muscular system. It was followed by a tranquillity extending so far as to procure sleep for three hours. The practice was not persisted in, and the child died." (P. 25.)

These cases deserve perusal, from the extreme minuteness with which they are detailed; and we are glad to learn that Mr. Pettigrew intends to publish a complete history of hydrophobia.

